# Ethyl 2-{[4-(pyridin-4-yl)pyrimidin-2-yl]sulfan­yl}acetate

**DOI:** 10.1107/S1600536811004272

**Published:** 2011-02-23

**Authors:** Chuan-Hu Wang

**Affiliations:** a Department of Applied Chemistry and Environmental Engineering, Bengbu College, Bengbu, 233030, People’s Republic of China

## Abstract

In the title mol­ecule, C_13_H_13_N_3_O_2_S, the pyridine and pyrimidine rings form a dihedral angle of 3.8 (1)°. The crystal packing exhibits weak inter­molecular C—H⋯O hydrogen bonds.

## Related literature

For details of the synthesis and general background to the rational design and assembly of coordination polymers with thio­ethers, see: Dong *et al.* (2008 ▶, 2009 ▶). For the crystal structures of coordination complexes with related ligands, see: Du *et al.* (2004 ▶); Zhu *et al.* (2009 ▶). 
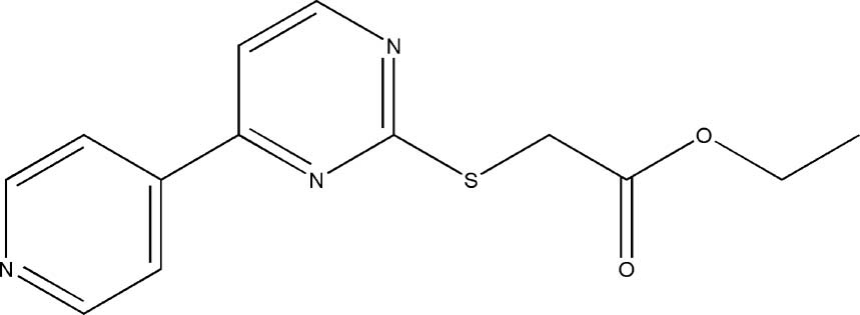


## Experimental

### 

#### Crystal data

C_13_H_13_N_3_O_2_S
*M* *_r_* = 275.33
Triclinic, 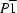

*a* = 8.6579 (8) Å
*b* = 9.7394 (9) Å
*c* = 9.9188 (8) Å
α = 62.661 (6)°
β = 71.416 (5)°
γ = 65.024 (6)°
*V* = 665.35 (10) Å^3^
*Z* = 2
Mo *K*α radiation
μ = 0.25 mm^−1^
*T* = 291 K
0.32 × 0.24 × 0.18 mm

#### Data collection

Bruker SMART CCD area-detector diffractometer
Absorption correction: multi-scan (*SADABS*; Bruker, 2000 ▶) *T*_min_ = 0.917, *T*_max_ = 0.966
11760 measured reflections
3021 independent reflections
2387 reflections with *I* > 2σ(*I*)
*R*_int_ = 0.032

#### Refinement

*R*[*F* ^2^ > 2σ(*F* ^2^)] = 0.038
*wR*(*F* ^2^) = 0.115
*S* = 1.04
3021 reflections
173 parameters
H-atom parameters constrained
Δρ_max_ = 0.24 e Å^−3^
Δρ_min_ = −0.20 e Å^−3^

### 

Data collection: *SMART* (Bruker, 2000 ▶); cell refinement: *SAINT* (Bruker, 2000 ▶); data reduction: *SAINT*; program(s) used to solve structure: *SHELXTL* (Sheldrick, 2008 ▶); program(s) used to refine structure: *SHELXTL*; molecular graphics: *SHELXTL*; software used to prepare material for publication: *SHELXTL*.

## Supplementary Material

Crystal structure: contains datablocks I, global. DOI: 10.1107/S1600536811004272/cv5047sup1.cif

Structure factors: contains datablocks I. DOI: 10.1107/S1600536811004272/cv5047Isup2.hkl

Additional supplementary materials:  crystallographic information; 3D view; checkCIF report

## Figures and Tables

**Table 1:** Hydrogen-bond geometry (Å, °)

*D*—H⋯*A*	*D*—H	H⋯*A*	*D*⋯*A*	*D*—H⋯*A*
C3—H3⋯O1^i^	0.93	2.52	3.383 (2)	154
C7—H7⋯O1^ii^	0.93	2.61	3.373 (2)	140

Symmetry codes: (i) 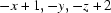; (ii) 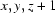.

**Fig. 1.**
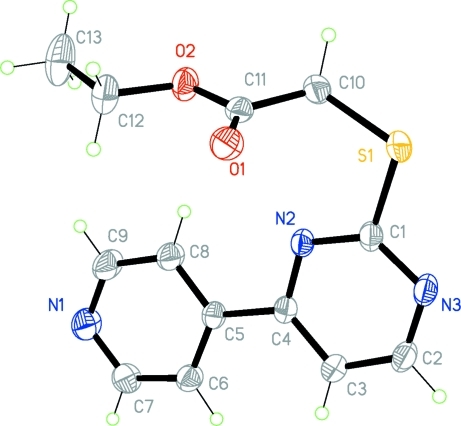
The structure of the title compound, showing 30% probability displacement ellipsoids and the atomic numbering.

## supplementary crystallographic information

### Comment

Remarkable attention has been paid to the rational design and assembly of new coordination polymers with thioethers (Dong *et al.*, 2008; 2009; Du *et al.*, 2004; Zhu *et al.*, 2009). Herewith we report the synthesis and crystal structure of the title compound (I)- a new derivative of 4-(4-pyridinyl)pyrimidine-2-thiol.

In (I) (Fig. 1), the pyridine and pyrimidine rings form a dihedral angle of 3.8 (1)°. The crystal packing exhibits weak intermolecular C—H···O hydrogen bonds (Table 1).

### Experimental

All solvents and chemicals were of analytical grade and were used without further purification. The title compound was prepared by similar procedure reported in the literature (Dong *et al.*, 2008; 2009), To a solution of 4-(4-pyridinyl)pyrimidine-2-thiol (3.78 g, 20 mmol) and sodium hydroxide (0.80 g, 20 mmol) in dry ethanol (100 ml), ethyl 2-bromoacetate (3.34 g, 20 mmol) in CCl_4_ (30 ml) was added. The mixture was stirred and refluxed for 8 h. After cooling, precipitates were filtered, washed by water and ethanol, and dried in vacuum. Single crystals suitable for X-ray diffraction were grown from methanol solution by slow evaporation in air at room temperature.

### Refinement

All H atoms were geometrically positioned (C—H 0.93–0.97 Å) and refined as riding, with *U*_iso_(H)=1.2–1.5 *U*_eq_ of the parent atom.

### Crystal data

**Table:**

C_13_H_13_N_3_O_2_S	*Z* = 2
*M**_r_* = 275.33	*F*(000) = 288.0
Triclinic, *P*1	*D*_x_ = 1.374 Mg m^−^^3^
Hall symbol: -P 1	Mo *K*α radiation, λ = 0.71073 Å
*a* = 8.6579 (8) Å	Cell parameters from 3021 reflections
*b* = 9.7394 (9) Å	θ = 2.3–27.5°
*c* = 9.9188 (8) Å	µ = 0.25 mm^−^^1^
α = 62.661 (6)°	*T* = 291 K
β = 71.416 (5)°	Block, colorless
γ = 65.024 (6)°	0.32 × 0.24 × 0.18 mm
*V* = 665.35 (10) Å^3^	

### Data collection

**Table:**

Bruker SMART CCD area-detector diffractometer	3021 independent reflections
Radiation source: fine-focus sealed tube	2387 reflections with *I* > 2σ(*I*)
graphite	*R*_int_ = 0.032
φ and ω scans	θ_max_ = 27.5°, θ_min_ = 2.3°
Absorption correction: multi-scan (*SADABS*; Bruker, 2000)	*h* = −11→10
*T*_min_ = 0.917, *T*_max_ = 0.966	*k* = −12→12
11760 measured reflections	*l* = −12→12

### Refinement

**Table:**

Refinement on *F*^2^	Primary atom site location: structure-invariant direct methods
Least-squares matrix: full	Secondary atom site location: difference Fourier map
*R*[*F*^2^ > 2σ(*F*^2^)] = 0.038	Hydrogen site location: inferred from neighbouring sites
*wR*(*F*^2^) = 0.115	H-atom parameters constrained
*S* = 1.04	*w* = 1/[σ^2^(*F*_o_^2^) + (0.0658*P*)^2^ + 0.067*P*] where *P* = (*F*_o_^2^ + 2*F*_c_^2^)/3
3021 reflections	(Δ/σ)_max_ < 0.001
173 parameters	Δρ_max_ = 0.24 e Å^−^^3^
0 restraints	Δρ_min_ = −0.20 e Å^−^^3^

### Special details

**Table:**

Experimental. The structure was solved by direct methods (Bruker, 2000) and successive difference Fourier syntheses.
Geometry. All e.s.d.'s (except the e.s.d. in the dihedral angle between two l.s. planes) are estimated using the full covariance matrix. The cell e.s.d.'s are taken into account individually in the estimation of e.s.d.'s in distances, angles and torsion angles; correlations between e.s.d.'s in cell parameters are only used when they are defined by crystal symmetry. An approximate (isotropic) treatment of cell e.s.d.'s is used for estimating e.s.d.'s involving l.s. planes.
Refinement. Refinement of *F*^2^ against ALL reflections. The weighted *R*-factor *wR* and goodness of fit *S* are based on *F*^2^, conventional *R*-factors *R* are based on *F*, with *F* set to zero for negative *F*^2^. The threshold expression of *F*^2^ > σ(*F*^2^) is used only for calculating *R*-factors(gt) *etc*. and is not relevant to the choice of reflections for refinement. *R*-factors based on *F*^2^ are statistically about twice as large as those based on *F*, and *R*- factors based on ALL data will be even larger.

### Fractional atomic coordinates and isotropic or equivalent isotropic displacement parameters (Å^2^)

**Table:**

	*x*	*y*	*z*	*U*_iso_*/*U*_eq_	
C1	0.21141 (18)	0.00339 (18)	0.89933 (17)	0.0411 (3)	
C2	0.3174 (2)	−0.21822 (19)	1.10105 (19)	0.0496 (4)	
H2	0.3517	−0.3308	1.1555	0.060*	
C3	0.3336 (2)	−0.12042 (18)	1.15846 (18)	0.0459 (4)	
H3	0.3793	−0.1652	1.2484	0.055*	
C4	0.27871 (17)	0.04721 (17)	1.07653 (16)	0.0376 (3)	
C5	0.28647 (18)	0.16653 (17)	1.12645 (16)	0.0388 (3)	
C6	0.3582 (2)	0.1176 (2)	1.25464 (18)	0.0478 (4)	
H6	0.4018	0.0070	1.3141	0.057*	
C7	0.3641 (2)	0.2347 (2)	1.2930 (2)	0.0568 (4)	
H7	0.4126	0.1991	1.3794	0.068*	
C8	0.2241 (2)	0.3332 (2)	1.04370 (19)	0.0525 (4)	
H8	0.1740	0.3725	0.9573	0.063*	
C9	0.2372 (3)	0.4407 (2)	1.0913 (2)	0.0660 (5)	
H9	0.1960	0.5521	1.0335	0.079*	
C10	0.0836 (2)	0.2913 (2)	0.6641 (2)	0.0515 (4)	
H10A	0.0315	0.3439	0.5718	0.062*	
H10B	−0.0042	0.3227	0.7439	0.062*	
C11	0.2273 (2)	0.35667 (18)	0.63290 (16)	0.0422 (3)	
C12	0.2788 (2)	0.5879 (2)	0.6083 (2)	0.0620 (5)	
H12A	0.3426	0.6012	0.5056	0.074*	
H12B	0.3603	0.5258	0.6805	0.074*	
C13	0.1774 (3)	0.7498 (3)	0.6187 (3)	0.0927 (8)	
H13A	0.1014	0.8124	0.5432	0.139*	
H13B	0.2542	0.8066	0.6002	0.139*	
H13C	0.1108	0.7354	0.7193	0.139*	
N1	0.3051 (2)	0.39441 (19)	1.21466 (18)	0.0644 (4)	
N2	0.21695 (15)	0.11055 (14)	0.94434 (14)	0.0392 (3)	
N3	0.25523 (17)	−0.15909 (16)	0.97173 (16)	0.0482 (3)	
O1	0.37818 (15)	0.29263 (14)	0.59842 (13)	0.0516 (3)	
O2	0.15904 (14)	0.50272 (13)	0.64426 (13)	0.0512 (3)	
S1	0.14603 (6)	0.07492 (5)	0.72151 (5)	0.05259 (16)	

### Atomic displacement parameters (Å^2^)

**Table:**

	*U*^11^	*U*^22^	*U*^33^	*U*^12^	*U*^13^	*U*^23^
C1	0.0408 (8)	0.0366 (8)	0.0520 (8)	−0.0153 (6)	−0.0068 (6)	−0.0197 (6)
C2	0.0542 (9)	0.0320 (8)	0.0601 (10)	−0.0176 (7)	−0.0095 (7)	−0.0119 (7)
C3	0.0514 (9)	0.0366 (8)	0.0489 (8)	−0.0168 (7)	−0.0111 (7)	−0.0113 (7)
C4	0.0365 (7)	0.0348 (7)	0.0428 (7)	−0.0142 (6)	−0.0023 (6)	−0.0159 (6)
C5	0.0402 (8)	0.0373 (8)	0.0415 (7)	−0.0142 (6)	−0.0015 (6)	−0.0188 (6)
C6	0.0573 (9)	0.0410 (8)	0.0463 (8)	−0.0143 (7)	−0.0124 (7)	−0.0162 (7)
C7	0.0713 (11)	0.0575 (11)	0.0533 (9)	−0.0186 (9)	−0.0167 (8)	−0.0282 (8)
C8	0.0712 (11)	0.0398 (9)	0.0521 (9)	−0.0135 (8)	−0.0201 (8)	−0.0192 (7)
C9	0.1000 (15)	0.0390 (9)	0.0678 (11)	−0.0152 (9)	−0.0285 (10)	−0.0238 (8)
C10	0.0549 (9)	0.0403 (8)	0.0677 (10)	−0.0094 (7)	−0.0271 (8)	−0.0213 (8)
C11	0.0533 (9)	0.0353 (8)	0.0405 (7)	−0.0108 (7)	−0.0175 (6)	−0.0130 (6)
C12	0.0637 (11)	0.0517 (10)	0.0791 (12)	−0.0294 (9)	−0.0024 (9)	−0.0277 (9)
C13	0.0989 (17)	0.0653 (14)	0.133 (2)	−0.0416 (13)	0.0127 (15)	−0.0589 (15)
N1	0.0889 (11)	0.0530 (9)	0.0664 (9)	−0.0200 (8)	−0.0205 (8)	−0.0321 (8)
N2	0.0434 (7)	0.0334 (6)	0.0460 (7)	−0.0143 (5)	−0.0076 (5)	−0.0172 (5)
N3	0.0534 (8)	0.0346 (7)	0.0629 (8)	−0.0176 (6)	−0.0102 (6)	−0.0199 (6)
O1	0.0513 (7)	0.0473 (7)	0.0572 (7)	−0.0104 (5)	−0.0108 (5)	−0.0241 (5)
O2	0.0521 (6)	0.0386 (6)	0.0680 (7)	−0.0148 (5)	−0.0100 (5)	−0.0237 (5)
S1	0.0670 (3)	0.0416 (2)	0.0651 (3)	−0.0161 (2)	−0.0253 (2)	−0.0246 (2)

### Geometric parameters (Å, °)

**Table:**

C1—N2	1.3323 (17)	C8—H8	0.9300
C1—N3	1.3367 (19)	C9—N1	1.332 (2)
C1—S1	1.7582 (16)	C9—H9	0.9300
C2—N3	1.329 (2)	C10—C11	1.514 (2)
C2—C3	1.383 (2)	C10—S1	1.7858 (16)
C2—H2	0.9300	C10—H10A	0.9700
C3—C4	1.387 (2)	C10—H10B	0.9700
C3—H3	0.9300	C11—O1	1.1984 (18)
C4—N2	1.3456 (18)	C11—O2	1.3342 (17)
C4—C5	1.4886 (19)	C12—O2	1.4529 (19)
C5—C8	1.385 (2)	C12—C13	1.479 (3)
C5—C6	1.388 (2)	C12—H12A	0.9700
C6—C7	1.381 (2)	C12—H12B	0.9700
C6—H6	0.9300	C13—H13A	0.9600
C7—N1	1.325 (2)	C13—H13B	0.9600
C7—H7	0.9300	C13—H13C	0.9600
C8—C9	1.385 (2)		
			
N2—C1—N3	127.86 (14)	C11—C10—S1	115.61 (11)
N2—C1—S1	118.93 (11)	C11—C10—H10A	108.4
N3—C1—S1	113.18 (10)	S1—C10—H10A	108.4
N3—C2—C3	123.15 (14)	C11—C10—H10B	108.4
N3—C2—H2	118.4	S1—C10—H10B	108.4
C3—C2—H2	118.4	H10A—C10—H10B	107.4
C2—C3—C4	117.32 (15)	O1—C11—O2	124.33 (14)
C2—C3—H3	121.3	O1—C11—C10	126.61 (14)
C4—C3—H3	121.3	O2—C11—C10	109.02 (13)
N2—C4—C3	120.88 (13)	O2—C12—C13	107.85 (15)
N2—C4—C5	116.22 (12)	O2—C12—H12A	110.1
C3—C4—C5	122.90 (13)	C13—C12—H12A	110.1
C8—C5—C6	116.98 (13)	O2—C12—H12B	110.1
C8—C5—C4	120.72 (13)	C13—C12—H12B	110.1
C6—C5—C4	122.29 (13)	H12A—C12—H12B	108.5
C7—C6—C5	119.32 (15)	C12—C13—H13A	109.5
C7—C6—H6	120.3	C12—C13—H13B	109.5
C5—C6—H6	120.3	H13A—C13—H13B	109.5
N1—C7—C6	124.28 (16)	C12—C13—H13C	109.5
N1—C7—H7	117.9	H13A—C13—H13C	109.5
C6—C7—H7	117.9	H13B—C13—H13C	109.5
C9—C8—C5	119.20 (15)	C7—N1—C9	116.14 (14)
C9—C8—H8	120.4	C1—N2—C4	116.09 (12)
C5—C8—H8	120.4	C2—N3—C1	114.65 (12)
N1—C9—C8	124.07 (17)	C11—O2—C12	116.28 (12)
N1—C9—H9	118.0	C1—S1—C10	101.64 (7)
C8—C9—H9	118.0		

### Hydrogen-bond geometry (Å, °)

**Table:**

*D*—H···*A*	*D*—H	H···*A*	*D*···*A*	*D*—H···*A*
C3—H3···O1^i^	0.93	2.52	3.383 (2)	154
C7—H7···O1^ii^	0.93	2.61	3.373 (2)	140

Symmetry codes: (i) −*x*+1, −*y*, −*z*+2; (ii) *x*, *y*, *z*+1.
